# 18F-FDG PET/CT Impact on Malignant Melanoma Patients Undergoing Staging and Restaging: A Single-University-Center Experience in a Real-World Setting

**DOI:** 10.3390/diagnostics15121560

**Published:** 2025-06-18

**Authors:** Silvija Lucic, Milena Spirovski, Borislava Nikolin, Dragana Stojanovic, Andrea Peter, Branislava Gajic, Vanja Cimbaljevic, Milos A. Lucic

**Affiliations:** 1 Medical Faculty, Oncology Institute of Vojvodina, University of Novi Sad, 21000 Novi Sad, Serbia; milena.spirovski@mf.uns.ac.rs (M.S.); borislava.nikolin@mf.uns.ac.rs (B.N.); andrea.peter@mf.uns.ac.rs (A.P.); milos.lucic@mf.uns.ac.rs (M.A.L.); 2 Oncology Institute of Vojvodina, 21000 Novi Sad, Serbia; draganadasa94@yahoo.com (D.S.); cimbaljevicvanja@gmail.com (V.C.); 3 Medical Faculty, University of Novi Sad, Clinic of Dermatovenereology Diseases, University Clinical Center of Vojvodina, 21000 Novi Sad, Serbia; branislava.gajic@mf.uns.ac.rs

**Keywords:** malignant melanoma, early stages, advanced stages, 18F-FDG PET/CT, survival

## Abstract

**Background/Objectives:** The global increase in the incidence of malignant melanoma, without significant changes in the mortality rate, may be influenced by the changes in the diagnostic approach and criteria, and the impact of innovative therapies on the survival of patients. Advances in treatment options, influencing prolonged survival, are bringing up a strong need for close surveillance of melanoma patients. In this observational, retrospective, and single-center study, we determined the impact of 18F-FDG PET/CT diagnostics on the outcomes and survival of malignant melanoma patients at different stages from an extensive and unselected group in a real-life clinical management setting. **Methods:** A total number of 189 malignant melanoma patients who underwent 18F-FDG PET/CT examination in the course of the treatment at one university oncology institute during the period from January 2010 to December 2024 were included in the study, and the multifactorial impact on the outcome and survival of malignant melanoma patients was observed in regard to the differences resulting from the therapeutic approaches and the introduction of new therapeutic options and drugs. **Results & Conclusions:** Our results indicate that 18F-FDG PET/CT is a sensitive imaging tool for the detection of predominantly distant metastases in malignant melanoma patients belonging to an extensive and unselected population in a real-world clinical setting, not only at advanced melanoma stages but also at early stages of high-risk patients’ surveillance. Follow-up appears to be of substantial importance for patients at advanced stages, but also for patients at early stages of disease, in particular in the presence of a strong clinical suspicion. Immunotherapy and combined therapy are improving overall survival in melanoma patients in real-world circumstances and equivalent clinical surroundings.

## 1. Introduction

The global increase in the incidence of malignant melanoma, without significant changes in the mortality rate, may be influenced by many factors, including the changes in the diagnostic approach and criteria, an increase in the number of biopsies, and the impact of innovative therapies on survival [1,2,3,4,5].

Malignant melanoma is the 17th most common tumor in the world with an ASR-W incidence of 3.2, and it was the 22nd-ranked tumor by mortality with an ASR-W mortality rate of 0.53 in 2020 [6].

In our country, melanoma was the 13th most common malignant tumor in 2022 and counted as the 17th most common malignant tumor in terms of mortality, with an increase in the share of 2.1% of all malignant tumors [7].

With surgery as the gold standard for early-stage treatment, no chemotherapy regimen has been shown to prolong survival in patients at advanced stages for decades. The development and introduction of new therapeutic modalities during the last decade in the treatment of metastatic melanoma significantly changed and improved the prognosis of these patients, influencing the improvement in five-year survival. Two main therapeutic options and their combinations, targeted therapy with BRAF and MEK inhibitors and immunotherapy with anti-CTLA and anti-PD-1, available in our country with their gradual introduction starting in December 2016, have brought about changes in the therapeutic approach but also improved the survival of patients with malignant melanoma [8,9,10].

Though a variety of different recommendations for 18F-FDG PET/CT are still present for malignant melanoma patients, there is strong evidence that, at advanced stages of melanoma (i.e., stages III and IV), FDG PET/CT can be of great value for the detection and localization of distant metastases, thereby influencing treatment decisions [11,12,13].

Potential indications for 18F-FDG PET/CT imaging extend across various melanoma stages and include primary staging after the initial presentation of the disease, surveillance in asymptomatic high-risk patients, preoperative evaluation of patients with metastatic disease who are candidates for surgery, and therapy effect monitoring [14].

The inclusion of 18F-FDG PET/CT into the oncologic diagnostic algorithm has certainly contributed to improving the clinical decision-making process [13,14], exerting an influence on more personalized and patient-specific therapeutic management strategies, but also melanoma patient outcomes and optimized survival rates.

Since the multifactorial impact on the outcome and survival of malignant melanoma patients in regard to the differences resulting from the therapeutic approaches and the introduction of new therapeutic options and drugs has been indicated [10,12,13], we have noted a limited amount of data in regard to the impact of 18F-FDG PET/CT on malignant melanoma patients’ outcomes and survival at different melanoma stages using data collected from an extensive and unselected group of patients in a real-world clinical setting.

In particular, we attempted to elucidate how 18F-FDG PET/CT influences the real-world clinical and diagnostic management of melanoma patients and discern the indicational decision-making process for its clinical use across different disease stages with the available therapeutical options.

Building on this, our research in this observational, retrospective, single-center study aimed to comprehensively assess the diagnostic value of 18F-FDG PET/CT imaging and its influence on the clinical outcomes and survival probabilities of malignant melanoma patients across different disease stages in a broad, unselected, and diverse group of patients managed in a real-world clinical setting with different available therapeutical options at a single university oncology institute during the period from January 2010 to December 2024.

## 2. Materials and Methods

### 2.1. Patients

The retrospective study design encompassed the collection and analysis of data from the hospital information system (HIS) and picture archiving and communication system (PACS) on melanoma patients that were diagnosed and treated in our University Center from January 2010 to December 2024. All patients underwent the 18F-FDG PET/CT imaging procedure during their disease course. We analyzed real-life clinical disease management conditions in relation to the performed 18F-FDG PET/CT diagnostic procedure and different available treatment options, and the outcome and survival were included in the analysis.

The study sample size was determined based on the population served by our hospital and an anticipated prevalence of 37.1 per 100,000 inhabitants. A margin of 5% was set, with the estimated confidence interval set between 85 and 90%. As a result, the required sample size was calculated to range between 163 and 199 patients.

Out of a total number of 219 patients identified through the HIS, based on the exclusion criteria (loss of follow-up, lack of follow-up for at least 12 months after 18F-FDG PET/CT, death within 12 months due to unknown causes, indication for 18F-FDG PET/CT related to another malignancy, inadequate diagnostic follow-up for the determination of outcome, and insufficient treatment information), 30 patients were excluded.

After the exclusion of 30 patients, representing 13.7% of the initial sample based on the exclusion criteria, and with a total number of 189 patients included in the study, we fulfilled the margin of error of 5.17% and, with 212 18F-FDG PET/CT examinations, the margin of error of 4.78%.

Finally, 189 patients fulfilled the inclusion criteria, having at least one 18F-FDG PET/CT diagnostic exam during staging and/or restaging, with a total number of 212 18F-FDG PET/CT examinations. The analysis was conducted on a per patient number (PPN) and per diagnostic scan number (PSN) basis. Patients were followed-up for at least 12 months, or until their death, and the final date of the follow-up evaluation was 31 December 2024.

Institutional ethical committee approval was obtained to access, collect, and analyze the data for the study.

Demographic data, the histopathologic evaluation regarding tumoral thickness (T), stage information, therapy treatment options, survival, and follow-up time were obtained from the hospital’s electronic medical records system (presented in Table 1).

Depending on the disease stage, patients were divided into two groups: a group with patients at stage I–II; and a group with patients at stage III–IV.

Additionally, based on the availability of innovative drugs, the following two time frame groups were defined: a group of patients who were treated prior to December 2016; and a group of patients treated after December 2016.

The accuracy of 18F-FDG PET/CT findings identified as positive or negative for the presence of malignant melanoma was classified as true positive (TP), true negative (TN), false positive (FP), or false negative (FN), based on up to 12 months (one year) of follow-up diagnostic exam results and/or validated histopathological results. The sensitivity, specificity, positive predictive value (PPV), negative predictive value (NPV), and diagnostic accuracy of 18F-FDG PET/CT exams were calculated.

Overall survival (OS) was calculated both as the time period from the initial diagnosis to the end of the study or the death of the patient and as the time period from the first 18F-FDG PET/CT exam to the end of the study or the death of the patient. Overall survival (OS) for the whole group, for the two staging groups, and for the two time frame groups was assessed.

Six patients, four with uveal and two with mucosal malignant melanoma, were excluded from the final statistical analysis of cutaneous-melanoma-specific OS (CMSOS).

### 2.2. Imaging Technique

18F-FDG PET/CT scans were acquired on two PET/CT scanners (Siemens Biograph 64, Erlangen, Germany and/or GE Discovery MI DR, Chicago, IL, USA). A whole-body PET scan was combined with low-dose CT from head to toes 60–90 min after the injection of 3.7 MBq/kg of 18F-FDG. Obligatory fasting for at least 4–6 h before examination and a glucose level in blood below 7 mmol/L (with the exception of diabetic patients, where the glucose level was below 11 mmol/L) were requested.

18F-FDG PET/CT scans were collected from the institutional PACS and blindly reviewed by radiologists and nuclear medicine specialists with more than five years of experience and a specific interest in oncology imaging.

### 2.3. Therapy Options

Treatment options for patients were determined based on the decision of the multidisciplinary oncology board, which was based on all relevant diagnostic and medical results for each patient. Until December 2016, available treatments included surgery, radiotherapy treatment, and chemotherapy regimens (DTIC–dacarbazine, CDDP/CCDP combined with cisplatin and/or carboplatin, or cisplatin + vinblastine + dacarbazine (CVD)). In addition to the listed therapy options, in December 2016 immunotherapy (nivolumab/pembrolizumab), targeted therapy (vemurafenib/dabrafenib), and combined targeted therapy (vemurafenib/dabrafenib + cobimetinib/trametinib) were introduced into the routine clinical treatment of malignant melanoma patients.

Since only a minor proportion of patients had the BRAF mutation, this feature was not analyzed and included in the results of the study.

### 2.4. Statistical Analysis

Statistical data analysis was performed using SPSS Statistics for Windows (version 22.0, IBM Inc., New York, NY, USA). Continuous variables are presented as the mean and median with ranges and categorical variables are presented as frequencies with percentages.

Descriptive statistics were used to report the demographic data, melanoma characteristics, treatment, and outcome. Two sample z-tests were used for categorical variable comparisons.

Deriving from the TPs, FPs, TNs, and FNs of routine 18F-FDG PET/CT imaging results, the diagnostic test sensitivity, specificity, PPV, NPV, and accuracy were calculated with 95% confidence intervals (CI) per patient and per diagnostic test number.

Kaplan–Meier survival curves and log-rank tests were used to analyze OS with medians and the 95% CI, comparing curves by the 18F-FDG PET/CT results, treatment time period outcome, and stage. Multivariable Cox regression analysis was used to estimate the hazard ratio, adjusted for 18F-FDG PET/CT, treatment time, and treatment type.

All statistical test results were two-sided, with a *p* value of <0.05 considered statistically significant.

## 3. Results

Demographic, clinical, and histopathologic data on the 189 included patients are presented in Table 1.

Staging after initial detection of newly diagnosed melanoma or clinical suspicion of recurrence in early-stage melanoma by 18F-FDG PET/CT was performed in 85 patients (with a mean and median time to exam of 5.95 ± 4.69; 3 months, range 1–12), and restaging after suspicion or detected local recurrence was performed in 104 patients (mean 49.09 ± 40.389, median 36 months, range 12–228).

**Figure 1:**
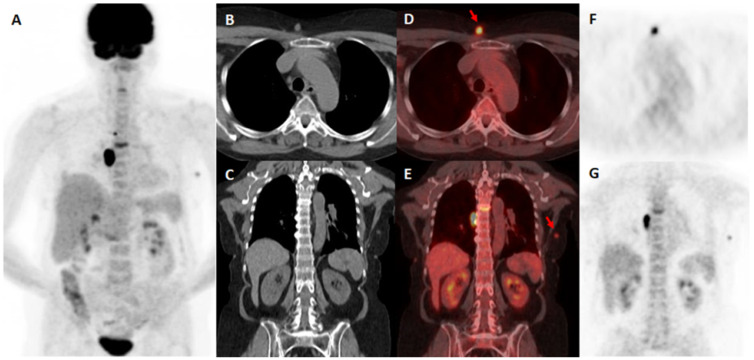
A 57-year-old female with exulcerated cutaneous melanoma of the right upper arm (Breslow 3.8 mm, T3b, stage II). 18F-FDG PET/CT was performed in order of restaging after histopathologically proven metastatic relapse. Positive 18F-FDG PET/CT with metastatic mediastinal involvement, subcutaneous metastases (red arrows), and thoracic spinal column infiltration. MIP 18F-FDG PET/CT (**A**). CT in the axial (**B**) and coronal (**C**) plane, fused PET/CT in the axial (**D**) and coronal (**E**) plane, and PET images in the axial (**F**) and coronal (**G**) plane.

**Figure 2:**
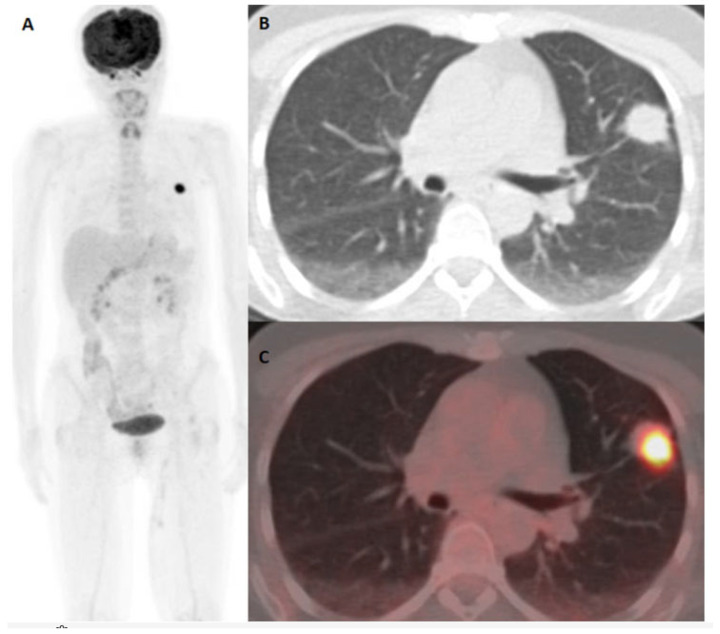
A 55-year-old female with cutaneous melanoma of the left thigh (Breslow 3.7 mm, pT3b, stage II). 18F-FDG PET/CT performed for staging purposes. Nodular lesion in the left lung proved to be an additional synchronous lung cancer. MIP 18F-FDG PET/CT shows a nodular lesion in the left lung (**A**). Axial chest CT (**B**) and fused PET/CT on the axial plane (**C**) demonstrate an 18F-FDG avid lesion in the left lung.

True-positive 18F-FDG PET/CT results indicated in the staging process are displayed in Figure 1 and in the process of restaging in Figure 2.

**Figure 3:**
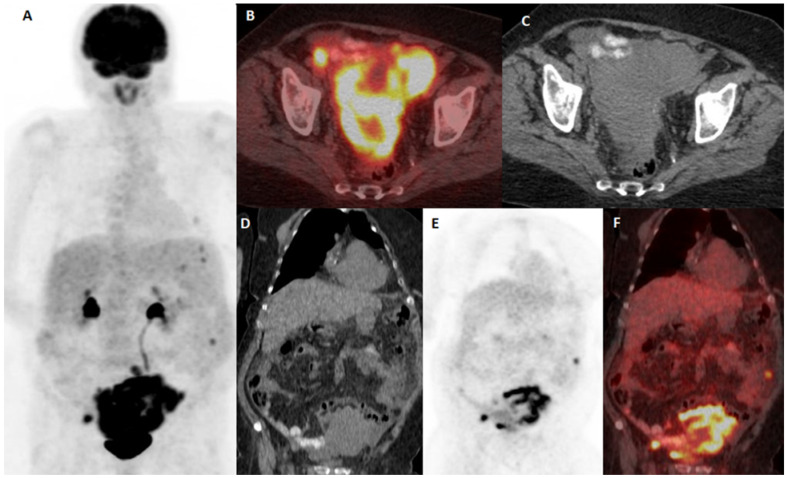
A 67-year-old female with cutaneous melanoma of the left inguinal region (Breslow V (4 mm), T3b, stage II). 18F-FDG PET/CT was done for restaging purposes after a clinical and US suspicion of recurrence. 18F-FDG PET/CT indicates metastatic involvement of intestine that proved to be a false positive due to a later histopathological biopsy finding of non-Hodgkin lymphoma. 18F-FDG PET/CT MIP shows abdominal and pelvic FDG avid lesions (**A**). Fused PET/CT (**B**) and CT (**C**) on the axial plane; CT on the coronal plane (**D**); PET (**E**) and fused PET/CT (**F**) on the coronal plane displaying FDG avidity within abdominal and pelvic intestine.

**Figure 4:**
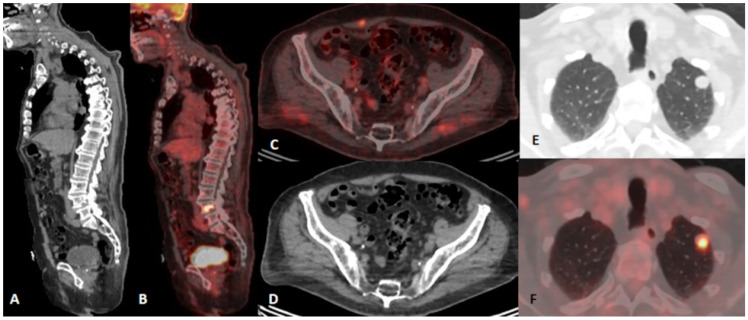
An 85-year-old male with left supraclavicular melanoma lymphonodal metastases of unknown origin. 18F-FDG PET/CT was performed for staging purposes after a possible metastatic lesion in the left lung was discovered on CT exam. 18F-FDG PET/CT indicative of metastatic involvement of the left lung and fourth lumbar vertebra and with an intramuscular lesion in the m. rectus abdominis. CT (**A**) and fused PET/CT (**B**) on the sagittal plane revealing metastasis in VL4; fused PET/CT (**C**) and CT (**D**) on the axial plane indicating metastasis in the abdominal wall. CT (**E**) and fused PET/CT (**F**) on the axial plane with an FDG avid metastatic lesion in the left lung.

Patients with false-positive 18F-FDG PET/CT results originating from the presence of an additional/concurrent primary tumor and metastatic cancer spread from an additional/concurrent tumor are presented in Figure 3 and Figure 4, respectively.

In the analysis of the 18F-FDG PET/CT results per diagnostic scan (total of 212 scans), three groups were discerned: initial staging and staging for recurrence after primary surgical treatment, consisting of 86 (40.56%) exams; restaging after locoregional and/or distal recurrence, numbering 107 (50.47%) exams; and a therapy evaluation group of 19 (9%) scans. The difference in the number of exams derives from the higher number of exams (23) indicated for recurrence suspicion (4 scans) or therapy follow-up (19 scans).

The sensitivity, specificity, and area under the curve (AUC) of 18F-FDG PET/CT were calculated per patient and per scan number and are presented in Table 2. The presented results are stratified by disease stage (stages I–II and stages III–IV) as well as by clinical indication, including staging, restaging, and therapy evaluation. Detailed diagnostic accuracy results for all diagnostic subgroups are provided in Table S1 in the Supplementary Material.

The calculated AUC values for all diagnostic groups, except for patients with stage III–IV disease and those undergoing 18F-FDG PET/CT for restaging, were greater than 0.85, indicating high classification accuracy. No statistically significant differences were observed between the calculated AUC values (*p*-value range: 0.59–0.99).

Brain metastases were detected in five TP exams, while in three TP exams 18F-FDG PET/CT failed to detect existing brain metastases (later detected by MRI). In four patients with TP results and three patients with FP results, brain metastases developed in further disease progression.

At the end of the observed period in the study, 65 out of 189 patients (34.4%) were alive, while 124 (65.6%) died. Of those, 118 (62.43%) died because of melanoma and six patients, or 3.17%, died due to other causes.

The overall survival (OS) from the initial diagnosis for melanoma-specific and cutaneous-melanoma-specific mortality is presented in Table 3. The OS from the time point of the 18F-FDG PET/CT exam, with 5- and 10-year survival rates, is presented in the Supplementary Material (Table S2).

The OS from initial diagnosis was 61 months for the entire study group (95% CI: 45.447–76.553), with no statistically significant difference in comparison to the melanoma-specific mortality (MSM) subgroup (95% CI: 45.547–74.453) and the cutaneous-melanoma-specific mortality (CMSM) subgroup (95% CI: 42.681–77.319) (*p* = 0.84148).

Statistically significant differences were observed between the OS of 132 months in patients with stage I and II disease (95% CI: 73.024–190.976) and 42 months in patients with stage III and IV disease (95% CI: 32.794–51.206) (*p* = 0.00006).

We did not find a statistically significant difference in OS between patients with stage I disease (179 months, 95% CI: 62.236–295.764) and stage II disease (132 months, 95% CI: 92.316–171.684) (*p* = 0.77182). Similarly, no significant difference was observed between stage III (39 months, 95% CI: 5.315–72.685) and stage IV patients (54 months, 95% CI: 41.559–66.441) (*p* = 0.27572).

A significant difference in OS was also observed between patients undergoing 18F-FDG PET/CT for staging (95% CI: 14.340–41.660) in comparison with those scanned for restaging (95% CI: 59.942–129.058) (*p* < 0.00001).

Patients with uveal melanoma and mucosal melanoma were excluded from the CMSM calculation. Still, we found that the median OS for uveal melanoma was 88 months (95% CI: 12.784–163.216).

**Figure 5:**
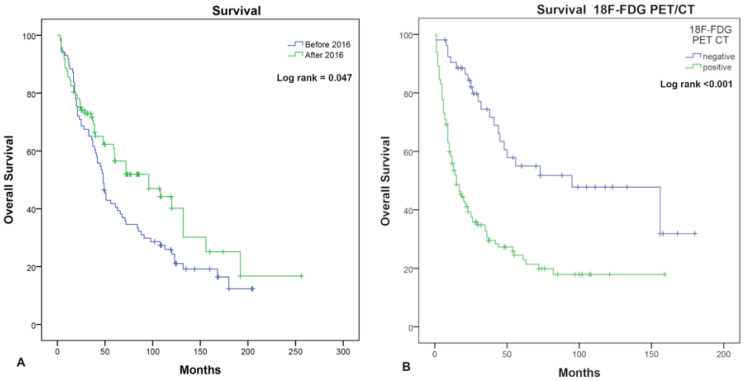
Kaplan–Meier OS (**A**) for two time frame groups and (**B**) for 18FDG PET/CT results.

In regard to the introduction of targeted therapy and immunotherapy, Kaplan–Meier analysis of OS from initial diagnosis showed a statistically significant difference before and after December 2016. Specifically, OS was 48 months (95% CI: 40.618–55.382) before December 2016 and 96 months (95% CI: 49.405–142.595) after December 2016 (Log-rank *p* = 0.047) for the duration of the study (Figure 5A).

Kaplan–Meier analysis of OS from the time of the 18F-FDG PET/CT exam time point revealed a significant difference between patients with negative and positive PET/CT results, where patients with negative 18F-FDG PET/CT scans had an OS of 95 months (95% CI: 27.965–162.035), compared with 15 months (95% CI: 9.681–20.319) for patients with positive scans (Log-rank *p* < 0.001), as presented in Figure 5B.

Kaplan–Meier analysis with the median survival time depending on the applied therapeutic modality is presented in the Supplementary Material (Figure S1).

**Figure 6:**
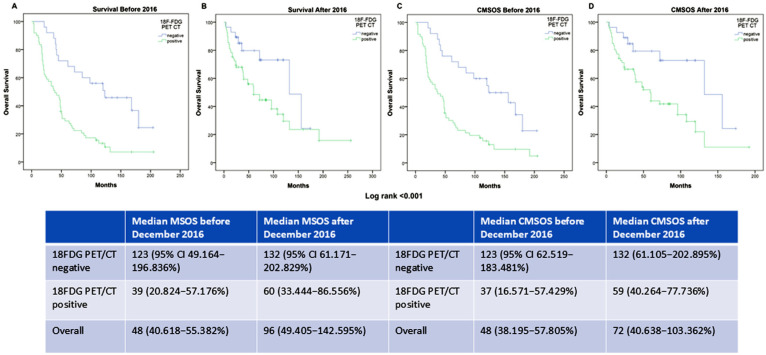
Kaplan–Meier analysis graphs showing melanoma-specific OS depending on 18F-FDG PET/CT findings for patients before (**A**) and after December 2016 (**B**) and cutaneous-melanoma-specific OS before (**C**) and after December 2016 (**D**) with the values presented in the table below. Abbreviations: MSOS—Melanoma-Specific Overall Survival; CMSOS—Cutaneous Melanoma-Specific Overall Survival.

The melanoma-specific OS and cutaneous-melanoma-specific overall survival (CMSOS) in relation to the 18F-FDG PET/CT results, before and after the introduction of targeted therapy and immunotherapy (December 2016), are presented in Figure 6.

Melanoma-specific OS (MSOS) and cutaneous-melanoma-specific overall survival (CMSOS), in relation to 18F-FDG PET/CT results, showed a statistically significant difference before and after the introduction of targeted therapy and immunotherapy in December 2016 (Log-rank *p* < 0.001).

In order to ascertain the effect of therapeutic modality, Cox proportional hazard regression was performed on 18F-FDG PET/CT results, stage of disease, time period before and after December 2016, age, and gender on survival. The model’s fit was evaluated with a −2Log Likelihood value of 1067.639, demonstrating statistical significance (*p* value < 0.05) as presented in Table 4.

## 4. Discussion

The continuous increase in the incidence of malignant melanoma worldwide with a rather stable mortality rate, together with the introduction of innovative therapies, has resulted in increased melanoma survivorship, leading to a greater need for disease surveillance [15].

The important role and great relevance of 18F-FDG PET/CT in the detection of distant metastases in staging, restaging, and therapy response assessment in patients with advanced stages of malignant melanoma (stage III and IV) have been recognized in a number of studies [16,17,18], while in early-stage melanoma (stage I–II) 18F-FDG PET/CT imaging is not routinely recommended [19,20].

Our results on diagnostic accuracy, especially considering sensitivity and specificity, are rather consistent with the results of El-Shourbagy et al., who found a sensitivity of 93.75%, a specificity of 60%, and a diagnostic accuracy of 84.21% for primary staging that differed across patients’ disease stages [21].

Zamani-Siahkali et al. reported slightly lower sensitivity (81%) but higher specificity (92%) in comparison with our results [22], while the study of Eldon et al. reported a lower sensitivity (82%), specificity (45%), and positive predictive value (22%) but a higher negative predictive value (93%) of 18F-FDG PET/CT in melanoma patients than we found [23].

In the meta-analysis of Xing et al., they reported a slightly lower sensitivity of 18F-FDG PET/CT (86%) but a higher specificity (91%) in the detection of distant metastases, but almost the same sensitivity and rather comparable specificity in the process of restaging and follow-up [24].

These differences may have arisen from the study’s construction, in our case based on real-world clinical management, with patients in different disease stages, consequently bringing about a higher number of false positive results. Several meta-analyses and prospective studies have established that FPs may vary between 1 and 25%, with limited data on distribution and various population types [25,26,27].

We believe that another possible cause of diagnostic accuracy variations is the number of false negative (FN) results, with the majority of them emerging from unidentified brain metastases due to the recognized lower 18F-FDG PET/CT sensitivity in brain metastasis detection [28].

A large number of melanoma patients in our study were in the unknown primary origin (Tx) group, and 39/53 (73.58%) had positive 18F-FDG PET/CT results. Of these, 79.07% were in the staging group and 62.5% were in the restaging group [23,29]. We believe that this result, which differs from the rather sparse available literature, could be explained by the fact that most of the patients in the Tx group in our study were already at stage IV, greatly increasing the proportion of positive results.

In the real-world circumstances, in the group of melanoma patients at stage I and II, where most of the patients were referred to an 18F-FDG PET/CT exam with a debatable clinical indication, we had 33.8% of all patients, with 31.13% of all 18F-FDG PET/CT exams conducted, and 53.03% positive results were obtained. Regardless of the different numbers of patients and different numbers of positive results in comparison with the results of the study done by Eldon et al., we found quite similar NPV values and slightly better sensitivity, specificity, and PPV values [23,30]. Interestingly, all of the patients with additional/concurrent primary tumors or metastatic cancer that spread from different tumors than melanoma were from this patient group. Even in the absence of a justified indication, the diagnostic value of 18F-FDG PET was evident. The introduction and implementation of national guidelines on the use of PET/CT in melanoma patients would definitely improve the diagnostic accuracy of 18F-FDG PET/CT in such realistic clinical settings.

Furthermore, in the stage I and II melanoma patient group, we were able to single out a subgroup of 23 patients in whom 18F-FDG PET/CT was performed within 12 months of initial diagnosis for two separate reasons: initial staging in patients with a T-thickness higher than 5 mm; and strong clinical suspicion of recurrence during follow-up.

The remaining 41 patients from the stage I and II group were referred to 18F-FDG PET/CT after histopathological verification of recurrence. Our study demonstrated higher sensitivity and specificity and a similar PPV to the study of Aviles Izquierdo et al., indicating a better NPV in particular, with a lower proportion of false positives (20.3%) [31].

Therefore, though it is affirmed that, without a strong clinical and/or diagnostic suspicion due to the higher rate of false positives at early melanoma stages, 18F-FDG PET/CT should not be recommended for routine surveillance [32], our results indicate that, in real-world clinical settings, an 18F-FDG PET/CT exam at the early melanoma stage may contribute to resolving the clinical suspicion.

Still, bearing in mind that 18F-FDG PET/CT overuse in low-risk, early-stage melanoma patients may lead to a notable proportion of false positive findings, 18F-FDG PET/CT in addition to negative psychological effects on patients can also lead to an increase in diagnostic overload and unnecessary costs. Therefore, we strongly accentuate that indications for 18F-FDG PET/CT should be carefully justified and based on well-supported clinical criteria.

The study of Frary et al. confirms that a high NPV (93.94%) at early melanoma stages suggests a good prognosis in the early stage of melanoma [33], but after a longer follow-up period (2 to 11 years), six patients with TN 18F-FDG PET/CT scan results had recurrences and died, which is in correlation with the Wagner et al. study [34].

It should be emphasized that, during follow-up within 12 months after 18F-FDG PET/CT, all of those six patients with FDG PET/CT TN findings had negative results on other diagnostic examinations (primarily US and CT).

On the other hand, in the same group, 22/64 (53.66%) patients died. Four patients had a TN 18F-FDG PET/CT exam and developed disease progression within 2–3 years. Based on our results, we share the opinion presented in other studies that, regardless of TN 18F-FDG PET/CT exams in patients at early melanoma stages, TN results and the high NPV value of FDG PET/CT should be taken as a short-term condition in a real-world clinical setting, and routine FDG PET/CT surveillance would be beneficial [35,36].

In almost half of the stage III and IV melanoma patients, 18F-FDG PET/CT was indicated after a histopathologically proven recurrence, with a median time of 36 months (range, 12–156 months). This implies the need for long-term FDG PET/CT surveillance (at least 5 years), which is in concordance with the results of other studies in the literature [37,38,39].

In our study, a large majority of 18F-FDG PET/CT exams were performed in the staging and/or restaging evaluation and were based on a real-world clinical indication of suspected distant metastatic disease, most frequently after ultrasound and/or MDCT diagnostic workup.

We found that melanoma recurrence occurred in 61% of patients, where the recurrence rate for stage II and stage III varied from 37% to 52% and the recurrence rate for stage IV was 90%, with a mean time of 36 months from diagnosis to recurrence, which is in accordance with other studies in the literature [39,40,41].

As presented in our results, 5-year and 10-year survival rates, as a factor of time from initial diagnosis or from 18F-FDG PET/CT, were generally a bit shorter than reported in the literature [42,43,44,45]. This has to be considered a multifactorial dependent feature.

Positive 18F-FDG PET/CT exam results showed an association with and strongly predicted a shorter OS than negative 18F-FDG PET/CT exam results in the whole group of patients and in the subgroups of patients treated before December 2016 and after December 2016. Evidently, new therapies are showing an important impact on melanoma patients’ survival. Patients treated with immunotherapy, targeted therapy, or combined targeted therapy with BRAF inhibitors had significantly better OS than patients treated with standard metastatic melanoma treatment options, e.g., local radiotherapy and/or chemotherapy, before December 2016.

Although there were no significant differences in median age between the two groups before and after December 2016, the point at which novel therapies were introduced, we acknowledge the possibility of selection bias, potentially coming from an unaccounted-for selection bias, where younger and fitter patients were more likely to receive newer treatment options, resulting in improved or more favorable OS outcomes.

The median OS for the 15-year duration of the study varied depending on the group of observed patients, from 132 months for the group of stage I–II melanoma patients to 42 months for the group of stage III–IV melanoma patients, affirming that melanoma patients’ survival varies strongly depending on the stage of the disease and the time since diagnosis, which we found to be in concordance with the results of other conducted and published studies [46,47].

Observed variations were stage specific, with a 5-year survival rate of 69% for patients at stage I and II to a survival rate of 41% for patients at stage III and IV. Differences in OS were observable in the primary staging group and the restaging after recurrence group, differing from 38% for staging to 64% for restaging.

Similarly to the results published by Malik et al. [48], our OS values were lower than those observed by other studies.

We determined that a positive 18F-FDG PET/CT exam resulted in significantly shorter OS throughout all study groups, indicating a statistically significant influence on OS and defining it as a relevant prognostic factor. This feature is in concordance with the results of other published studies [38,48], indicating that a positive 18F-FDG PET/CT has a significant impact on patients’ OS.

We observed significant differences in OS between patients treated with immunotherapy and targeted therapy in favor of immunotherapy and combined targeted therapy. These differences match the findings in OS described by Crispo et al. [49]. Understanding that disease progression during therapy with a BRAF inhibitor alone could be rapid and unresponsive to subsequent treatments, a reduction in rapid progression has been achieved with the addition of MEK inhibitors [49,50].

Additionally, the worse OS in patients treated with targeted therapy may emanate from the fact that patients with poorer prognostic factors were treated with targeted therapy in expectation of a more rapid response [51], but we must declare that this conclusion and the potential debate reach beyond the scope of our study.

Multivariable hazard ratio covariates indicate that disease stage and 18F-FDG PET/CT results were significant predictors of survival, while therapy options depending on the time period, before and after December 2016, were on the borderline of statistical significance.

When adjusting for different treatment options, Cox regression analysis pointed out that disease stage and all treatment options (with the exception of chemotherapy) significantly influence OS. In the adjusted data set analysis, we found 18F-FDG PET/CT to be on the very border of statistical significance, while age and gender did not demonstrate any significance as a predictor of survival.

Several limitations influenced our study. The relatively small number of 18F-FDG PET/CT exams performed on melanoma patients was probably caused by the lack of national guidelines for PET/CT indications and a regulatory rule that requires prerequisite diagnostic exams to implement immunotherapy and/or targeted therapies in melanoma patients and does not include 18F-FDG PET/CT as a diagnostic imaging modality that can be requested for therapy approval.

Still, we believe that the main limitation of this study is the relatively incoherent sample of patients, which may have resulted from the inclusion criteria, including the time frame in which 18F-FDG PET/CT was performed on melanoma patients treated at our university hospital, resulting in diversity in the sample caused by real-world circumstances. The number of patients with different T-thicknesses, stages, and indications for 18F-FDG PET/CT may have influenced the diagnostic boundaries of this imaging test. The broad range of disease and patient attributes that we faced in the real-world clinical setting have indubitably contributed to a higher number of false-positive but also false-negative FDG PET/CT results, influencing the study results.

Inconsistent specific disease characteristics in patients, accompanied by the wide and inhomogeneous range of available treatment strategies, divided by the time of their implementation, before and after the introduction of novel therapy options, did not statistically disturb the significant difference in OS before and after December 2016.

## 5. Conclusions

Our study results indicate that 18F-FDG PET/CT is a sensitive imaging tool for the detection of predominantly distant metastases in malignant melanoma patients belonging to an extensive and unselected population in a real-world clinical setting, not only at advanced melanoma stages but also at early stages in high-risk patients. Follow-up is of substantial importance to both patients at advanced stages and patients at early stages of disease, particularly in the presence of a strong clinical suspicion.

18F-FDG PET/CT exam results manifest a strong connection with melanoma patients’ prognosis and survival, providing information that may serve as a predictive biomarker of outcome and OS in melanoma patients.

Our results show that immunotherapy and combined therapy do improve overall survival in melanoma patients in real-world circumstances and equivalent clinical surroundings.

Further studies that include melanoma patients treated with immunotherapy and targeted therapies are needed to determine the more profound impact on survival. We believe that uncoupling a patient group treated with those therapies in an adjuvant setting would be of importance.

It is our belief that the introduction of consistent national guidelines on imaging would have a strong impact on appropriate, optimal, and standardized use of the 18F-FDG PET/CT modality in sustainable and maximally personalized malignant melanoma patient care in both real-world and strict clinical settings.

## Figures and Tables

**Table 1:** Demographic, histopathologic, clinical, diagnostic, and follow-up data on patients’ features.

Characteristics	Number
Gender (No)	189
Men	103 (54.5%)
Women	86 (45.5%)
Tumoral thickness, intervals in mm (T) (No)	185
T1 (a + b)	1 + 6 (0.5 + 3.2%)
T2 (a + b)	6 + 21 (3.2 + 11.1%)
T3 (a + b)	17 + 38 (9 + 20.1%)
T4 (a + b)	6 + 37 (3.2 + 19.6%)
Tx (unknown primary)	53 (28%)
AJCC stage (No)	185
I	5 (2.6%)
II	59 (31.2%)
III	15 (7.9%)
IV	106 (56.1%)
Treated before December 2016	90 (47.6%)
Treated after December 2016	99 (52.4%)
Age at diagnosis (mean, median, range)	53.22 ± 14.62; 54; 19–88
Age at 18F-FDG PET/CT (mean, median, range)	55.67 ± 14.28; 57; 21–88
Melanoma type:	
Cutaneous	183 (96.82%)
Uveal	4 (2.12%)
Mucosal	2 (1.06%)
Folow-up (mean, median, range)	65.5 ± 56.41; 48; 3–312 months
Follow-up time from 18F-FDG PET/CT exam (mean, median, range)	35.14 ± 39.16 21; 1–180 months
Time frame from initial diagnosis until 18F-FDG PET/CT (mean, median, range)	29.34 ± 37.07; 12; 1–228 months
Outcome	
Dead	124 (65.6%)
Alive	65 (34.4%)
18F-FGD PET/CT per patient/per scan	
Negative	56 (29.6%)/63 (29.7%)
Positive	133 (70.4%)/149 (70.4%)
True positive	96/109
False positive	32/35
True negative	53/60
False negative	8/8

**Table 2:** 18F-FDG PET/CT sensitivity, specificity, and AUC test results for the whole group of patients, diagnostic scan numbers, stage I and II group, and stage III and IV group and separate indications for group staging and restaging.

	Per Patient Number	Per Scan Number	Stage I and II	Stage III and IV	Staging Indication Group	Restaging Indication Group	Therapy Evaluation Indication Group
Sensitivity (95%CI)	92.38% (85.54–96.65%)	93.22% (87.08–97.03%)	90.48% (69.62–98.83%)	92.59% (84.57–97.23%)	90.24% (76.87–97.28%)	93.65% (84.53–98.24%)	92.31% (63.97–99.81%)
Specificity (95%CI)	61.9% (50.66–72.29%)	62.77% (52.18–72.52%)	69.77% (53.87–82.82%)	50% (33.38–66.62%)	68.18% (52.42–81.39%)	56.1% (39.75–71.53%)	83.33% (35.88–99.58%)
AUC * (95% CI)	0.86 (0.714–0.942)	0.865 (0.779–0.949)	0.872 (0.801–0.942)	0.819 (0.713–0.926)	0.865 (0.792–0.938)	0.846 (0.748–0.944)	0.9261 (0.878–0.974)

Abbreviation: * AUC—area under the curve.

**Table 3:** Overall survival (OS) for melanoma-specific and cutaneous-melanoma-specific mortality from the initial diagnosis.

	Whole Group (*n* = 189)	MSM * (*n* = 183)	CMSM ** (*n* = 177)	Stage I and II (*n* = 64)	Stage III and IV (*n* = 121)	18F-FDG PET/CT Staging (*n* = 84)	18F-FDG PET/CT Restaging (*n* = 94)
OS from initial diagnosis (months) (95% CI)	61 (45.447–76.553)	60 (45.547–74.453)	60 (42.681–77.319)	132 (73.024–190.976)	42 (32.794–51.206)	28 (14.340–41.660)	94 (59.942–129.058)

Abbreviations: * MSM, melanoma-specific mortality; ** CMSM, cutaneous-melanoma-specific mortality (uveal and mucosal melanoma excluded).

**Table 4:** Non-adjusted and adjusted Cox regression analysis results.

	Therapy		Time Point (Before/After December 2016)		18FDG PET/CT		Stage		Age		Gender	
*p* value	0.098		0.068		0.0001		0.002		0.82		0.914	
HR (95% CI for Exp(B))	0.905 (0.805–1.018)		0.699 (0.475–1.027)		2.659 (1.627–4.346)		1.331 (1.107–1.6)		1.002 (0.988–1.016)		1.021 (0.702–1.484)	
	No Th	Radio Th	Chemo Th	Immuno Th	Targeted Th	Combined Th	Surgery	Time point	FDG	Stage	Age	Gender
*p* value	0.00001	0.00001	0.081	0.001	0.013	0.05	0.01	0.243	0.081	0.017	0.206	0.604
Adjusted HR 95% CI for Exp(B)	0.206 (0.093–0.457)	/	0.521 (0.25–1.084)	0.218 (0.09–0.527)	0.37 (0.168–0.814)	0.23 (0.083–0.639)	0.241 (0.101–0.574)	0.757 (0.475–1.208)	1.633 (0.941–2.834)	1.251 (1.041–1.504)	1.01 (0.995–1.025)	1.107 (0.754–1.624)

Abbreviations: No Th—no therapy; Radio Th—radiotherapy; Chemo Th—chemotherapy; Immuno Th—immunotherapy; Targeted Th—targeted therapy; Combined Th—combined therapy; Time Point—time point before/after December 2016; FDG—18F-FDG PET/CT.

## Data Availability

The data that support the findings of this study are available from the corresponding author (S.L.) upon reasonable request.

## Supplementary Materials

The following supporting information can be downloaded at: https://www.mdpi.com/article/10.3390/diagnostics15121560/s1, Table S1: 18F-FDG PET/CT diagnostic test results for the whole group of patients, diagnostic scan numbers, stage group I & II, stage group III & IV, and separately indications for group staging and restaging; Table S2: Overall survival (OS) for the melanoma specific and cutaneous melanoma specific mortality from 18F-FDG PET/CT exam time, with 5- and 10-year survival rates from initial diagnosis and from 18F-FDG PET/CT time point; Figure S1: Kaplan-Meier analysis graph showing melanoma specific OS depending on therapy modality, with median OS in months presented in table below for each aplied therapy modality.
