# Supplementary material for: 18F-FDG PET/CT Impact on Malignant Melanoma Patients Undergoing Staging and Restaging: A Single-University-Center Experience in a Real-World Setting
Source: Diagnostics (Basel). 2025 Jun 18;15(12):1560. doi: 10.3390/diagnostics15121560 (PMC12192031; doi:10.3390/diagnostics15121560)
Supplement: Supplementary file 1 [file diagnostics-15-01560-s001.zip › diagnostics-3648515-supplementary.pdf]

Table 1. 18F-FDG PET/CT diagnostic test results for the whole group of patients, diagnostic scan numbers, stage group I & II, stage group III & IV, and separately indications for group staging and restaging

	Sensitivity (95%CI)	Specificity (95%CI)	PPV (95%CI)	NPV (95%CI)	Accuracy (95%CI)	Disease prevalence
Per patient number	92.38% (85.54-96.65%)	61.9% (50.66-72.29%)	75.19% (69.65-80.01%)	86.67% (76.58-92.82%)	78.84% (72.32-84.43%)	55.56% (48.17-62.77%)
Per scan number	93.22% (87.08-97.03%)	62.77% (52.18-72.52%)	75.68% (70.64-80.41%)	88.06% (78.77-93.61%)	79.72% (73.67-84.91%)	55.66% (48.7-62.46%)
Stage I & II	90.48% (69.62-98.83%)	69.77% (53.87-82.82%)	59.38% (47.62-70.15%)	93.75% (79.82-98.27%)	76.56% (64.31-86.25%)	32.81% (21.59-45.69%)
Stage III & IV	92.59% (84.57-97.23%)	50% (33.38-66.62%)	79.79 (74.06-84.51%)	76% (57.92-87.93%)	78.99% (70.57-85.92%)	68.07% (58.9-76.31%)
Staging indication group	90.24% (76.87-97.28%)	68.18% (52.42-81.39%)	72.55 (62.9-80.47%)	88.24% (74.29-95.11%)	78.82% (68.61-86.94%)	48.24 (37.26-59.34)
Restaging indication group	93.65% (84.53-98.24%)	56.1% (39.75-71.53%)	76.62% (69.75-82.33%)	85.19% (68.2-93.91%)	78.85% (69.74-86.24%)	60.58 (50.51-70.02)
Therapy evaluation indication group	92.31% (63.97-99.81%)	83.33% (35.88-99.58%)	92.31% (66.57-98.64%)	83.33% (42.38-97.14%)	89.47% (66.86-98.75%)	68.42% (43.45-87.42%)

Table 2. Overall survival (OS) for the melanoma specific and cutaneous melanoma specific mortality from 18F-FDG PET/CT exam time, with 5- and 10-year survival rates from initial diagnosis and from 18F-FDG PET/CT time point.

	5-year survival rate (%)	10-year survival rate (%)	OS from 18F-FDG PET/CT (months)	5-year survival rate (%)	10-year survival rate (%)
Whole group (N=189)	51±0.64	35±0.91	25 (95% CI 15.433-34.567)	33±0.42	24±0.7
MSM* (N=183)	51±1.38	34±1.47	26 (95% CI 16.086-35.914)	33±0.82	27±1.3
CMSM** (N=177)	50±1.38	34±1.49	26 (95% CI 15.761-36.239)	33±0.82	26±1.28
Stage I & II (N=64)	69±2.58	54±3.13	61 (95% CI 0.0-123.0.38)	56±2.09	44±2.54
Stage III & IV (N=121)	41±1.25	16±0.76	23 (95% CI 11.826-34.174)	29±0.79	15±0.59
18F-FDG PET/CT staging (N=84)	38±1.26	27±1.47	18 (95% CI 7.726-28.274)	35±1.21	26±1.42
18F-FDG PET/CT restaging (N=94)	64±0.39	38±2.03	73 (95% CI 5.963-140.037)	30±0.9	23±1.19

Abbreviations: *MSM - melanoma specific mortality; **CMSM - cutaneous melanoma specific mortality (uveal and mucosal melanoma excluded)

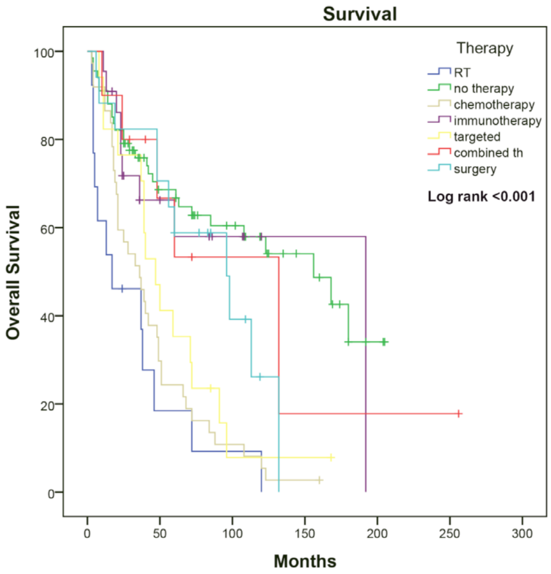

**Patients** (N=183)	**No therapy** (months)	**RT (radiotherapy)** (months)	**Chemotherapy** (months)	**Immuno therapy** (months)	**Targeted therapy** (months)	**Combined therapy** (months)	**Surgery** (months)	**Overall survival** (months)
**Median OS**	156 (95% CI 92.431-219.569)	17 (95% CI 0.0-50.028)	36 (95% CI 19.315-52.685)	192 (/)	47 (95% CI 32.210-61.790)	132 (95% CI 79.295-184.705)	96 (95% CI 45.174-146.826)	60 (95% CI 45.547-74.453)

Figure 1. Kaplan-Meier analysis graph showing melanoma specific OS depending on therapy modality, with median OS in months presented in table below for each aplied therapy modality.
